# Optimal Computational Power Allocation in Multi-Access Mobile Edge Computing for Blockchain

**DOI:** 10.3390/s18103472

**Published:** 2018-10-15

**Authors:** Yuan Wu, Xiangxu Chen, Jiajun Shi, Kejie Ni, Liping Qian, Liang Huang, Kuan Zhang

**Affiliations:** 1 College of Information Engineering, Zhejiang University of Technology, Hangzhou 310023, China; 2111703318@zjut.edu.cn (X.C.); 2111703412@zjut.edu.cn (J.S.); 2111703062@zjut.edu.cn (K.N.); lpqian@zjut.edu.cn (L.Q.); lianghuang@zjut.edu.cn (L.H.); 2 State Key Laboratory of Integrated Services Networks, Xidian University, Xi’an 710071, China; 3 Department of Electrical and Computer Engineering, University of Nebraska-Lincoln, Omaha, NE 68182-0572, USA; kzhang22@unl.edu

**Keywords:** multi-access, mobile edge computing, computational power allocation, optimization, Blockchain

## Abstract

Blockchain has emerged as a decentralized and trustable ledger for recording and storing digital transactions. The mining process of Blockchain, however, incurs a heavy computational workload for miners to solve the proof-of-work puzzle (i.e., a series of the hashing computation), which is prohibitive from the perspective of the mobile terminals (MTs). The advanced multi-access mobile edge computing (MEC), which enables the MTs to offload part of the computational workloads (for solving the proof-of-work) to the nearby edge-servers (ESs), provides a promising approach to address this issue. By offloading the computational workloads via multi-access MEC, the MTs can effectively increase their successful probabilities when participating in the mining game and gain the consequent reward (i.e., winning the bitcoin). However, as a compensation to the ESs which provide the computational resources to the MTs, the MTs need to pay the ESs for the corresponding resource-acquisition costs. Thus, to investigate the trade-off between obtaining the computational resources from the ESs (for solving the proof-of-work) and paying for the consequent cost, we formulate an optimization problem in which the MTs determine their acquired computational resources from different ESs, with the objective of maximizing the MTs’ social net-reward in the mining process while keeping the fairness among the MTs. In spite of the non-convexity of the formulated problem, we exploit its layered structure and propose efficient distributed algorithms for the MTs to individually determine their optimal computational resources acquired from different ESs. Numerical results are provided to validate the effectiveness of our proposed algorithms and the performance of our proposed multi-access MEC for Blockchain.

## 1. Introduction

Blockchain, a distributed and trustable architecture for recording and storing digital transactions, has been considered as one the promising mechanisms for enabling the secure cyber-physical systems [1]. In the framework of Blockchain, the miners participate in a mining game [2], and all miners compete with each other to be the first winner to solve the proof-of-work puzzle (which corresponds to executing a series of hashing computation). After solving the proof-of-work puzzle and broadcasting the mined block to other miners to reach the consensus, the winner can claim the consequent reward. Nowadays, there exist several different mining pools (e.g., Slush Pool and AntPool), in which the miners can select to join and participate in the mining game/process [3]. Thanks to the nature of distributed management and independence from the central authorities, Blockchain has been expected to play a crucial role as a distributed and trustable ledger for recording and storing a variety of transactions and resource exchange, e.g., the crypto-currency and peer-to-peer electronic payment system [4], energy trading in smart grid [5,6], computation offloading in vehicular networks [7], radio resource exchange in wireless networks [8], and Internet of Things (IoTs) [9,10,11,12].

However, solving the proof-of-work puzzle requires consuming a significant amount of computational resources, which are prohibitive from the perspective of the mobile terminals (MTs). To address this difficulty, the MTs can exploit the recent advanced mobile edge computing (MEC) to enhance their computational capability [13,14]. In particular, the MEC enables the MTs to offload their computational workloads to the edge servers (ESs) equipped with sufficient computational resources and deployed at the edge of the radio access networks (e.g., the cellular base stations, BSs), which thus reduces the computation time and improves the resource utilization efficiency. As a result, MEC has been considered as one of the enabling technologies for realizing the future IoTs and the fifth generation (5G) visions which aim at providing massive connectivity, high access speed, and low latency [15]. The recent multi-access MEC, which can be enabled by the advanced Non-orthogonal multiple access (NOMA) [16,17,18], further allows the MTs to acquire computational resources from several ESs simultaneously, and thus yields a flexible exploitation of the computational resources from multiple ESs [19,20,21]. Therefore, by integrating MEC into the framework of Blockchain, the computational capability of the MTs can be effectively enhanced, which thus facilitates solving the proof-of-work puzzle and increases the successful probability in winning the mining game [22,23].

Thanks to its advantage, MEC has been exploited in many different paradigms, e.g., green IoT and vehicular networks [24,25,26]. In the following, we mainly review the studies about the resource management for MEC. Focusing on the single user’s binary task-offloading, Melendez et al. proposed the offloading decisions for reducing the completion time [27]. Further taking into account the radio resource utilization in MEC, many studies have been devoted for the joint optimization of task offloading and radio resource allocation [28,29,30]. Optimal offloading strategies for partial computation offloading (in which the MTs are allowed to offload partial of their computational workloads) have been investigated in [31,32]. The scenario of multi-user MEC is close to our study here. Focusing on minimizing the delay in completing the computation tasks, there have been many studies investigating the proper multi-user computational resource allocation [33,34]. In [35], a multi-user computational resource allocation (i.e., the CPU cycle) scheme has been proposed to save the users’ energy consumption. In [36], Jin et al. proposed an auction-based scheme for sharing the computational resource at the mobile edge. In [37], a revenue-maximization oriented computational resource allocation scheme has been proposed. In particular, some recent work investigated the exploitation of MEC for Blockchain. In [22], Xiong et al. exploited the MEC for Blockchain and investigated the resource management and pricing strategy. In particular, the paper formulated a two-stage Stackelberg game to maximize the profit of the edge service provider and the utilities of the miners. In [23], Liu et al. studied the computation offloading and content caching in wireless Blockchain networks with MEC. In particular, the economics based mechanisms have been envisioned as the promising approaches for enabling efficient yet distributed resource allocations in wired or wireless networks [38,39,40], in which the pricing mechanism and game theoretic modelling have been widely exploited. For instance, in [41], Ha et al. proposed time dependent pricing scheme for mobile data management. In [42], Tsiropoulou et al. proposed a novel joint customized price and power control for optimizing the energy-efficiency in multi-service wireless networks. In recent work [43], by taking into account the competitive wireless Internet service market, Vamvakas et al. proposed an efficient pricing strategy for the joint dynamic provider selection and the associated power allocation. In this work, we exploit the mechanism of the dual price (which is also referred as the shadow price) to achieve an efficient allocation of the edge servers’ computational resources to different mobile users/miners.

In this work, we investigate the optimal allocation of the computational resource/power in multi-access MEC enabled Blockchain. As described before, the multi-access MEC enables each MT to obtain the computational power from multiple ESs simultaneously (in the following, we use the term of computational power and the term of computational resource interchangeably). As a result, the MT can increase its successful probability when participating in the mining game and gain the consequent reward (i.e., winning the bitcoin). However, as a compensation to the ESs, the MT needs to pay for the consequent cost for acquiring the computational resources. Our contributions in this work can be summarized as follows:

- To investigate the trade-off between obtaining the computational resources from the ESs (for solving the proof-of-work) and paying for the consequent cost, we focus on a scenario in which a group of the MTs acquire the computational powers from a set of nearby ESs and pay for the consequent costs for acquiring the computational powers. Mathematically, we formulate an optimization problem in the MTs to determine their acquired computational powers from different ESs, with the objective of maximizing the MTs’ total net-reward while keeping the fairness among the MTs.
- Despite the non-convexity of the formulated optimization problem, we exploit its layered structure and design two algorithms (one for the one-ES scenario and the other for the multi-ES scenario) to find the optimal solution efficiently. We also provide extensive numerical results to validate the effectiveness of our proposed algorithms and show the performance of our proposed multi-access MEC for Blockchain.

The reminder of this paper is organized as follows. We present the system model and problem formulation in Section 2. We first focus on the one-ES scenario and propose a distributed algorithm to compute the optimal solution in Section 3. We then consider the multi-ES scenario and propose a corresponding distributed algorithm to achieve the optimal solution in Section 4. We conclude this work in Section 5 and discuss the future directions.

## 2. System Model and Problem Formulation

### System Model

**Figure 1:**
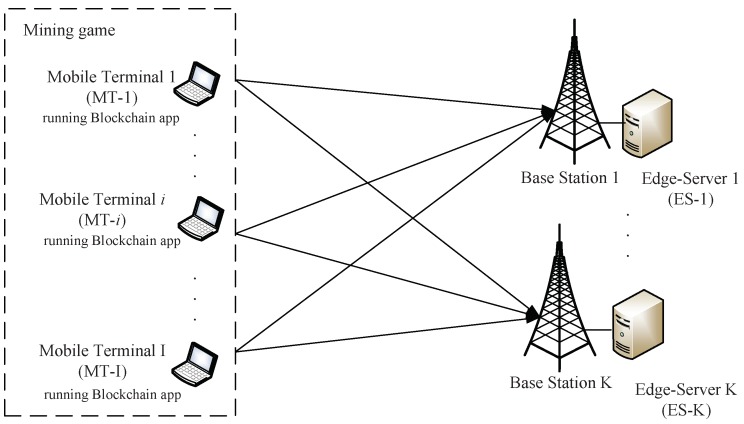
System model: a group of mobile terminals offload computational workloads to the edge servers.

We consider a system model as shown in Figure 1. Specifically, a group of the ESs denoted by K={1,2,…,K} provide the computing services to a group of the MTs which are denoted by I={1,2,…,I}. Enabled by the multi-access MEC, each MT i∈I can acquire the computational power from the ESs simultaneously. Specifically, we use $x_{i}^{k}$ to denote MT *i*’s computational power from ES *k* and use $x_{i}^{\mathrm{loc}}$ to denote MT *i*’s local computational power. Thus, each MT *i*’s total computational power $\theta_{i}$ can be expressed as:

(1) $$\begin{array}{c} \theta_{i}=x_{i}^{\mathrm{loc}}+\sum_{k\in K}x_{i}^{k},\forall i\in I. \end{array}$$

In this work, $\theta_{i}$, $x_{i}^{\mathrm{loc}}$, and $x_{i}^{k}$ are all measured in the unit of GHash/sec. Furthermore, we introduce $\alpha_{i}$ to denote MT *i*’s total computational power with respect to the overall computational power of all MTs, i.e.,

(2) $$\begin{array}{c} \alpha_{i}=\frac{\theta_{i}}{\sum_{i\in I}\theta_{i}}=\frac{x_{i}^{\mathrm{loc}}+\sum_{k\in K}x_{i}^{k}}{\sum_{i\in I}x_{i}^{\mathrm{loc}}+\sum_{i\in I}\sum_{k\in K}x_{i}^{k}}. \end{array}$$

In the mining game, the MTs compete against each other to be the first one to solve the proof-of-work puzzle and receive the reward accordingly. Similar to [22], we model the successful probability that MT *i* wins the mining game (including that MT *i* successfully mines the block and its solution reaches the consensus) as a random variable as follows:

(3) $$\begin{array}{c} P_{i}\left(\alpha_{i},t_{i}\right)=\alpha_{i}\left(1-P_{orphan}\left(t_{i}\right)\right), \end{array}$$

where $t_{i}$ denotes MT *i*’s block-size, and function $P_{orphan}\left(t_{i}\right)$ denotes the orphaning probability [22] as follows:

(4) $$\begin{array}{c} P_{orphan}\left(t_{i}\right)=1-e^{-\lambda t_{i}}. \end{array}$$

The use of the above $P_{orphan}\left(t_{i}\right)$ can be explained as follows. After solving the proof-of-work puzzle, MT *i* needs to broadcast its result to other MTs for reaching the consensus. Since the broadcasting of the computation-result among the MTs suffers from a certain delay, it is possible that MT *i* fails to be the first one whose computation-result reaches the consensus among all MTs (even though that MT *i* is the first one who solves the proof-of-work puzzle). The orphaning probability $P_{orphan}\left(t_{i}\right)$ in (4) quantifies such a probability. In particular, the same as [22,44], we express $P_{\left(t_{i}\right)}$ as the Poisson distribution with parameter λ, in which parameter λ denotes the inter-arrival rate of the Poisson distribution. Based on (3), we can express MT *i*’s net-reward function in winning the mining game as follows:

(5) $$\begin{array}{c} m_{i}=\left(R+rt_{i}\right)P_{i}\left(\alpha_{i},t_{i}\right)-\sum_{k\in K}p^{k}x_{i}^{k}, \end{array}$$

where *R* denotes the fixed reward, and $rt_{i}$ denotes the variable reward which linearly grows with MT *i*’s block-size $t_{i}$ (parameter *r* is a fixed constant). Parameter $p^{k}$ denotes the marginal price of ES *k* for providing the computational power to MT *i*.

In this work, we consider that the MTs acquire the computational power from the group of the ESs with the objective of maximizing the total net-reward, while keeping the fairness among them. To this end, we formulate the following total net-reward optimization (TRO) problem:

(6) $$\begin{array}{cc} (\mathrm{TRO}):\; & \max\sum_{i\in I}\ln\left(m_{i}\right),\\ \mathrm{subject}\;\mathrm{to}:\; & \mathrm{constraints}\;(1),(2),(3),\;\mathrm{and}\;(5),\\ & 0\leq \sum_{i\in I}x_{i}^{k}\leq C^{k,\mathrm{tot}},\forall k\in K,\\ \mathrm{variables}:\; & x_{i}^{k}\geq 0,\forall i\in I,k\in K. \end{array}$$

Constraint (6) ensures that all MTs’ total computational power acquired from ES *k* cannot exceed ES *k*’s maximum computational power $C^{k,\mathrm{tot}}$. Notice that since both $\theta_{i}$ and $\alpha_{i}$ depend on ${\left\{x_{i}^{k}\right\}}_{k\in K}$, we just treat ${\left\{x_{i}^{k}\right\}}_{i\in I,k\in K}$ as the decision variables in Problem (TRO). However, Problem (TRO) is a complicated non-convex optimization problem, and there exists no general algorithm that can solve it efficiently [45]. We will propose a distributed algorithm to compute the optimal solution in the next two sections. Specifically, in our proposed algorithm, each MT individually determines the acquired computational powers from different ESs. Then, viewing the aggregate demands from all MTs, the ESs further update their respective computational powers allocated to all MTs for maximizing the total net-reward. Thus, our algorithm does not require a central entity to collect the global information in the considered network. Nevertheless, the downside of our proposed algorithm is that it requires the message exchange between the MTs and the ESs, meaning that the MTs and ESs need to take the additional burdens on sending and receiving the required messages for reaching the optimal solution.

## 3. One-ES Scenario and Proposed Distributed Algorithm

### 3.1. Problem Formulation and Its Decomposition

We first consider one-ES scenario and aim at finding the optimal allocation of the computational resource for the MTs. For the sake of easy presentation, we use ES k=1 as an example in the following. In particular, with one ES, Problem (TRO) turns into :

$$\begin{array}{cc} (\mathrm{TRO-ES}):\; & \max\sum_{i\in I}\ln\left(\left(R+rt_{i}\right)\frac{x_{i}^{\mathrm{loc}}+x_{i}^{1}}{\sum_{i\in I}x_{i}^{\mathrm{loc}}+\sum_{i\in I}x_{i}^{1}}e^{-\lambda t_{i}}-p^{1}x_{i}^{1}\right),\\ \mathrm{subject}\;\mathrm{to}:\; & 0\leq \sum_{i\in I}x_{i}^{1}\leq C^{1,\mathrm{tot}},\\ \mathrm{variables}:\; & x_{i}^{1}\geq 0,\forall i\in I. \end{array}$$

However, Problem (TRO-ES) is still a non-convex optimization problem, which is difficult to solve in general. To efficiently solve Problem (TRO-ES), we adopt a vertical decomposition as follows. We firstly introduce an auxiliary variable μ to denote all MTs’ total computational power obtained from ES 1, i.e.,

(7) $$\begin{array}{c} \sum_{i\in I}x_{i}^{1}=\mu , \end{array}$$

with $0\leq \mu \leq C^{1,\mathrm{tot}}$.

Suppose that the value of μ is given in advance. We thus aim at solving the following subproblem:

$$\begin{array}{cc} (\mathrm{TRO-ES-Sub}):\; & V_{\mu }^{\mathrm{sub}}=\max\sum_{i\in I}\ln\left(A_{i}x_{i}^{1}+B_{i}\right), \end{array}$$

(8) $$\begin{array}{cc} \mathrm{subject}\;\mathrm{to}:\; & \sum_{i\in I}x_{i}^{1}=\mu , \end{array}$$

(9) $$\begin{array}{cc} & A_{i}x_{i}^{1}+B_{i}\geq 0,\forall i\in I,\\ \mathrm{variables}:\; & x_{i}^{1}\geq 0,\forall i\in I, \end{array}$$

with parameters $A_{i}$ and $B_{i}$ given by:

(10) $$\begin{array}{ccc} A_{i} & = & \frac{\left(R+rt_{i}\right)e^{-\lambda t_{i}}}{\sum_{i\in I}x_{i}^{\mathrm{loc}}+\mu }-p^{1}, \end{array}$$

(11) $$\begin{array}{ccc} B_{i} & = & \frac{\left(R+rt_{i}\right)e^{-\lambda t_{i}}}{\sum_{i\in I}x_{i}^{\mathrm{loc}}+\mu }x_{i}^{\mathrm{loc}}. \end{array}$$

Notice that, in Subproblem (TRO-ES-Sub), we use $V_{\mu }^{\mathrm{sub}}$ to denote the optimal value of Subproblem (TRO-ES-Sub), which depends on the given value of μ (i.e., the total computational power obtained from ES 1).

After solving Problem (TRO-ES-Sub) and obtaining $V_{\mu }^{\mathrm{sub}}$ (for each given μ), we continue to find the optimal value of μ (denoted by $\mu^{*}$) for maximizing $V_{\mu }^{\mathrm{sub}}$, i.e., solving the following top-problem:

$$\begin{array}{cc} (\mathrm{TRO-ES-Top}):\; & \max V_{\mu }^{\mathrm{sub}},\\ \mathrm{variable}:\; & 0\leq \mu \leq C^{1,\mathrm{tot}}. \end{array}$$

The reason for us to adopt the above proposed vertical decomposition is as follows. Given the value of μ, Subproblem (TRO-ES-Sub) is a strictly convex optimization (i.e., Proposition 1 provided below), which enables us to solve it efficiently. In the next subsection, we propose a distributed algorithm to solve Subproblem (TRO-ES-Sub) and Top-problem (TRO-ES-Top).

### 3.2. Proposed Algorithm to Solve Subproblem (TRO-ES-Sub)

To efficiently solve Subproblem (TRO-ES-Sub), we firstly identify the following property.

**Proposition** **1.** *Given μ, Subproblem (TRO-ES-Sub) is a strictly convex optimization.*

**Proof.**  Given the value of μ, the values of ${\left\{A_{i},B_{i}\right\}}_{i\in I}$ are all fixed. Thus, according to the convex optimization theory [45], Problem (TRO-ES-Sub) is a strictly convex optimization problem. ☐

The convexity of Subproblem (TRO-ES-Sub) enables us to use the Karush–Kuhn–Tucker (KKT) conditions [45] to compute the optimal solution. In particular, to solve Subproblem (TRO-ES-Sub), we identify the following three possible cases.

*Case I* in which we have $A_{i}<0,\forall i\in I$, and $\underset{i\in I}{\sum }\left(-\frac{B_{i}}{A_{i}}\right)\geq \mu$.

In Case I, we define $C_{i}=-A_{i},\forall i\in I$. With ${\left\{B_{i},C_{i}\right\}}_{i\in I}$, Problem (TRO-ES-Sub) can be equivalently changed into Problem (TRO-ES-Sub-I):

(12) $$\begin{array}{cc} (\mathrm{TRO}-\mathrm{ES}-\mathrm{Sub}-I):\; & V_{\mu }^{\mathrm{sub}}=\max\sum_{i\in I}\ln\left(B_{i}-C_{i}x_{i}^{1}\right),\\ \mathrm{subject}\;\mathrm{to}:\; & \sum_{i\in I}x_{i}^{1}\geq \mu ,\\ \mathrm{variables}:\; & 0\leq x_{i}^{1}\leq \frac{B_{i}}{C_{i}},\forall i\in I. \end{array}$$

Problem (TRO-ES-Sub-I) is again a strictly convex optimization problem. Moreover, it can be observed that constraint (12) is strictly binding at the optimum. Thus, we introduce the dual variable λ to relax (12) and obtain the Lagrangian function as (where the subscript “I” stands for Case I):

(13) $$\begin{array}{c} L_{I}\left({\left\{x_{i}^{1}\right\}}_{i\in I},\lambda \right)=\sum_{i\in I}\ln\left(B_{i}-C_{i}x_{i}^{1}\right)+\lambda \left(\sum_{i\in I}x_{i}^{1}-\mu \right). \end{array}$$

With the KKT condition and (13), we can derive the optimal solution for Problem (TRO-ES-Sub-I) as follows:

(14) $$\begin{array}{c} x_{i}^{1*}=\max\left\{\frac{B_{i}}{C_{i}}-\frac{1}{\lambda^{*}},0\right\},\forall i\in I, \end{array}$$

where $\lambda^{*}$ is determined according to the following condition:

(15) $$\begin{array}{c} \sum_{i\in I}x_{i}^{1*}=\mu . \end{array}$$

Based on (14) and (15), we can propose the following distributed algorithm (i.e., Algorithm 1) to solve Problem (TRO-ES-Sub-I). Notice that, in Algorithm 1, exploiting the monotonic property of (14), we use the bisection-search (i.e., from Step 3 to Step 11) to find $\lambda^{*}$ until condition (15) is satisfied.

**Algorithm 1:** To solve Problem (TRO-ES-Sub-I) and determine ${\left\{x_{i}^{1}\right\}}^{*}$ and $V_{\mu }^{\mathrm{sub}}$**Input**: Each MT *i*’s $A_{i}$ and $B_{i}$. **Initialization**: ES 1 sets $\bar{\lambda }$ as a sufficiently large number and $\underline{\lambda }=0$. Set the tolerance for the computational error ε as a small number. **while**
$|\bar{\lambda }-\underline{\lambda }|>\varepsilon$
**do** ES 1 sets $\lambda^{\mathrm{cur}}=\frac{\bar{\lambda }+\underline{\lambda }}{2}$ and broadcasts $\lambda^{\mathrm{cur}}$ to all MTs. Each MT i∈I sets $x_{i}^{1}=\max\left\{\frac{B_{i}}{C_{i}}-\frac{1}{\lambda^{\mathrm{cur}}},0\right\}$ and reports $x_{i}^{1}$ to ES 1. **if**
$\underset{i\in I}{\sum }x_{i}^{1}>\mu$
**then**  ES 1 updates $\bar{\lambda }=\lambda^{\mathrm{cur}}$. **else**  ES 1 updates $\underline{\lambda }=\lambda^{\mathrm{cur}}$. **end if** **end while** ES 1 sets $\lambda^{*}=\lambda^{\mathrm{cur}}$ and broadcasts $\lambda^{*}$ to all MTs in I. Each MT i∈I sets $x_{i}^{1*}=\max\left\{\frac{B_{i}}{C_{i}}-\frac{1}{\lambda^{*}},0\right\}$ and reports the value of $\ln(A_{i}x_{i}^{1*}+B_{i})$ to ES 1. **Output**: ES 1 outputs $V_{\mu }^{\mathrm{sub}}=\sum_{i\in I}\ln\left(B_{i}-C_{i}x_{i}^{1*}\right)$ based on all MTs’ reports.

*Case II* in which there exists a subset of MT i∈I with $A_{i}>0$. In particular, we denote this subset as $I_{\mathrm{sub}}=\left\{i\in I|A_{i}>0\right\}$. 

In Case II, we can derive the optimal solution of Problem (TRO-ES-Sub) as follows. For each MT *i* with $A_{i}<0$, we set $x_{i}^{*}=0$ directly. For the MTs in $I_{\mathrm{sub}}$, we can express Problem (TRO-ES-Sub) as:

(16) $$\begin{array}{cc} (\mathrm{TRO-ES-Sub-II}):\; & V_{\mu }^{\mathrm{sub}}=\max\sum_{i\in I_{\mathrm{sub}}}\ln\left(A_{i}x_{i}^{1}+B_{i}\right),\\ \mathrm{subject}\;\mathrm{to}:\; & \sum_{i\in I_{\mathrm{sub}}}x_{i}^{1}\leq \mu ,\\ \mathrm{variables}:\; & x_{i}^{1}\geq 0,\forall i\in I_{\mathrm{sub}}. \end{array}$$

It can be observed that Subproblem (TRO-ES-Sub-II) is a strictly convex optimization problem, and the optimal solution occurs when constraint (16) is binding. We thus introduce the dual variable λ to relax (16) and obtain the following Lagrangian function:

(17) $$\begin{array}{c} L_{\mathrm{II}}\left({\left\{x_{i}^{1}\right\}}_{i\in I},\lambda \right)=\sum_{i\in I_{\mathrm{sub}}}\ln\left(A_{i}x_{i}^{1}+B_{i}\right)+\lambda \left(\mu -\sum_{i\in I_{\mathrm{sub}}}x_{i}^{1}\right). \end{array}$$

With the KKT condition and (17), we can derive the optimal solution for Subproblem (TRO-ES-Sub-II) as follows:

(18) $$\begin{array}{c} x_{i}^{1*}=\max\left\{\frac{1}{\lambda^{*}}-\frac{B_{i}}{A_{i}},0\right\},\forall i\in I_{\mathrm{sub}}, \end{array}$$

with $\lambda^{*}$ determined according to the following condition

(19) $$\begin{array}{c} \sum_{i\in I_{\mathrm{sub}}}x_{i}^{1*}=\mu . \end{array}$$

Based on (18) and (19), we can propose the following distributed algorithm (i.e., Algorithm 2) to solve Problem (TRO-ES-SubII). Notice that, in Algorithm 2, exploiting the monotonic property of (18), we use the bisection-search (i.e., from Step 4 to Step 12) to find $\lambda^{*}$ until condition (19) is satisfied.

**Algorithm 2:** To solve Problem (TRO-ES-Sub-II) and determine $\left\{x_{i}^{1*}\right\}$ and $V_{\mu }^{\mathrm{sub}}$**Input**: Each MT *i*’s $A_{i}$ and $B_{i}$. **Initialization**: ES 1 sets $\bar{\lambda }$ as a sufficiently large number and $\underline{\lambda }=0$. Set the tolerance for the computational error ε as a small number. MT i∈I sets $x_{i}^{1*}=0$ if its $A_{i}\leq 0$ and reports to ES 1. **while**
$|\bar{\lambda }-\underline{\lambda }|>\varepsilon$
**do** ES 1 sets $\lambda^{\mathrm{cur}}=\frac{\bar{\lambda }+\underline{\lambda }}{2}$ and broadcasts $\lambda^{\mathrm{cur}}$ to the MTs in $I_{\mathrm{sub}}$. Each MT $i\in I_{\mathrm{sub}}$ sets $x_{i}^{1}=\max\left\{\frac{1}{\lambda^{\mathrm{cur}}}-\frac{B_{i}}{A_{i}},0\right\}$ and reports $x_{i}^{1}$ to ES 1. **if**
$\underset{i\in I}{\sum }x_{i}^{1}>\mu$
**then**  ES 1 updates $\underline{\lambda }=\lambda^{\mathrm{cur}}$. **else**  ES 1 updates $\bar{\lambda }=\lambda^{\mathrm{cur}}$. **end if** **end while** ES *i* sets $\lambda^{*}=\lambda^{\mathrm{cur}}$ and sends $\lambda^{*}$ to all MTs in $I_{\mathrm{sub}}$. Each MT $i\in I_{\mathrm{sub}}$ sets $x_{i}^{1*}=\max\left\{\frac{1}{\lambda^{\mathrm{cur}}}-\frac{B_{i}}{A_{i}},0\right\}$ and reports the value of $\ln(A_{i}x_{i}^{1*}+B_{i})$ to ES 1. **Output**: ES 1 outputs $V_{\mu }^{\mathrm{sub}}=\sum_{i\in I_{\mathrm{sub}}}\ln\left(A_{i}x_{i}^{1*}+B_{i}\right)+\sum_{i\in I,i\notin I_{\mathrm{sub}}}\ln\left(B_{i}\right)$ based on all MTs’ reports.

*Case III* in which we have $A_{i}<0,\forall i\in I$ and moreover, $\underset{i\in I}{\sum }\left(-\frac{B_{i}}{A_{i}}\right)<\mu$. Case III is a trivial case in which Problem (TRO-ES-Sub) is infeasible. 

As a summary of the above Case I, Case II, and Case III, we propose the following Algorithm 3 to solve Problem (TRO-ES-Sub) and determine ${\left\{x_{i}^{1*}\right\}}_{i\in I}$ and $V_{\mu }^{\mathrm{sub}}$. In Algorithm 3, we use Algorithm 1 (in Step 7) and Algorithm 2 (in Step 10) as the subroutines. Until now, we have completed solving Problem (TRO-ES-Sub) for the given value of μ.

**Algorithm 3:** To solve Problem (TRO-ES-Sub) and determine ${\left\{x_{i}^{1}\right\}}^{*}$ and $V_{\mu }^{\mathrm{sub}}$**Input**: The value of μ. Each MT i∈I reports its $x_{i}^{\mathrm{loc}}$ to ES 1. ES 1 computes $X^{\mathrm{loc}}=\sum_{i\in I}x_{i}^{\mathrm{loc}}$ and sends $X^{\mathrm{loc}}$ to all MTs. Each MT *i* uses (10) to compute $A_{i}=\frac{\left(R+rt_{i}\right)e^{-\lambda t_{i}}}{X^{\mathrm{loc}}+\mu }-p^{1}$, and uses (11) to compute $B_{i}=\frac{\left(R+rt_{i}\right)e^{-\lambda t_{i}}}{X^{\mathrm{loc}}+\mu }x_{i}^{\mathrm{loc}}$. Each MT reports its $\left(A_{i},B_{i}\right)$ to ES 1. **if** Case I holds **then** Use Algorithm 1 to output ${\left\{x_{i}^{1*}\right\}}_{i\in I}$ and $V_{\mu }^{\mathrm{sub}}$. **else** **if** Case II holds **then**  Use Algorithm 2 to output ${\left\{x_{i}^{1*}\right\}}_{i\in I}$ and $V_{\mu }^{\mathrm{sub}}$. **else**  Output that Problem (TRO-ES-Sub) is infeasible under the current μ. **end if** **end if**

### 3.3. Proposed Algorithm to Solve Top-Problem (TRO-ES-Top)

We continue to solve Top-problem (TRO-ES-Top) in this subsection. Notice that, for each given μ, we can use Algorithm 3 to obtain the value of $V_{\mu }^{\mathrm{sub}}$. However, the difficulty in solving Top-problem (TRO-ES-Top) lies in that we cannot derive $V_{\mu }^{\mathrm{sub}}$ analytically. As a result, Top-problem (TRO-ES-Top) is an optimization problem in which the objective function cannot be analytically given, which thus prevents us from using the conventional gradient based approach to solve it. Fortunately, the viable interval of the μ is fixed, namely, $\mu \in [0,C^{1,\mathrm{tot}}]$. Exploiting this property, we can use a linear-search (LS) with a very small step-size to numerically find the best value of μ (which is denoted by $\mu^{*}$) that can maximize $V_{\mu }^{\mathrm{sub}}$. The details are shown in the following Algorithm 4. Notice that, in Step 3, we use Algorithm 3 as the subroutine to determine the value of $V_{\mu }^{\mathrm{sub}}$ (and the corresponding optimal ${\left\{x_{i}^{1*}\right\}}_{i\in I}$) under the currently enumerated μ.

**Algorithm 4:** To solve Top-problem (TRO-ES-top) and determine $\mu^{*}$**Initialize**: CBV=0 and CBS=∅. Set Δ as a very small step-size. **for**
$\mu =\Delta :\Delta :C^{1,\mathrm{tot}}$
**do** Use Algorithm 3 to determine $V_{\mu }^{\mathrm{sub}}$ and ${\left\{x_{i}^{1*}\right\}}_{i\in I}$. **if**
$V_{\mu }^{\mathrm{sub}}>\mathrm{CBV}$
**then**  Set $\mathrm{CBS}={\left\{x_{i}^{1*}\right\}}_{i\in I}$ and $\mathrm{CBV}=V_{\mu }^{\mathrm{sub}}$. **end if** **end for** **Output**: Set the optimal solution according to CBS.

The computational complexity of our Algorithm 4 can be analyzed as follows. First, as the internal subroutines of Algorithm 4, both Algorithms 1 and 2 require executing a bisection search with the complexity of $\log_{2}\left(\frac{\bar{\lambda }-\underline{\lambda }}{\epsilon }\right)$. In addition, the linear search in Algorithm 4 requires consuming $\frac{C_{1}^{\mathrm{tot}}}{\Delta }$ rounds of the iterations (notice that only one of the two subroutines, i.e., Algorithms 1 and 2, will be invoked in each round of the iterations). As a result, the total complexity of our proposed Algorithm 4 is given by $\frac{C_{1}^{\mathrm{tot}}}{\Delta }\log_{2}\left(\frac{\bar{\lambda }-\underline{\lambda }}{\epsilon }\right)$.

### 3.4. Numerical Results for One-ES Scenario

We present the numerical results to validate the effectiveness of our proposed algorithms to solve Problem (TRO-ES). We set the parameter-settings according to the data provided in [46] (Table 1 lists the detailed settings). Specifically, we set $\lambda =\frac{1}{600}$ (i.e., the average generating-time for each block is 10 min) and each block-size $t_{i}=1$ Mbit. Meanwhile, according to [46], we set R=7000$ for each block and set r=1000 $/Mbit. In addition, for MT *i*, the local computational power $x_{i}^{k}$ is randomly generated according to a uniform distribution within [1,2] GHash/sec. Finally, we set $p^{1}=10\;$$/GHash according to the ES’s unit cost for providing the computational power.

**Figure 2:**
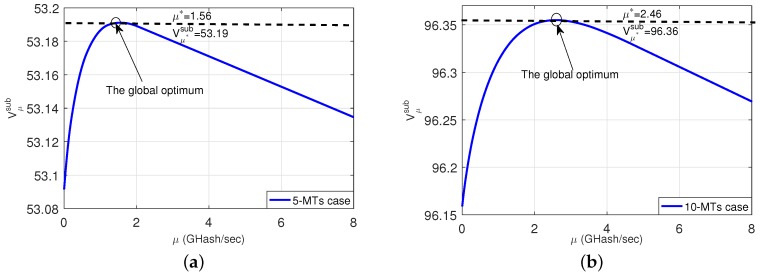
$V_{\mu }^{\mathrm{sub}}$ versus different μ. (**a**): a 5-MT case; (**b**): a 10-MT case.

To illustrate the rationale of our proposed decomposition, we provide Figure 2 to show how $V_{\mu }^{\mathrm{sub}}$ varies with different μ. We test a 5-MT case (in the left subplot) and a 10-MT case (in the right subplot). Figure 2 shows that $V_{\mu }^{\mathrm{sub}}$ firstly increases when μ increases, and then gradually decreases when μ is beyond a certain threshold. Such a phenomenon of $V_{\mu }^{\mathrm{sub}}$ matches the intuition very well, namely, neither a too small μ nor too small μ will be beneficial to the computation offloading. On the one hand, when μ is too small, the MTs can only obtain a small amount of computational power from the ES, which results in a small total net-reward. On the other hand, when μ is too large, a large cost is incurred for obtaining the computational power, which again degrades the total net-reward. This phenomenon is the motivation of our work, i.e., to find the optimal trade-off between exploiting the computational power provided by the ES and the consequent cost.

Table 2 validates the effectiveness of our Algorithm 4 for solving Top-problem (TRO-ES-Top). For the purpose of comparison, we use a benchmark scheme that exploits the convexity of Problem (TRO-ES-Sub). Specifically, we use CVX [45] (which is a commercial solver for convex optimization) to solve Problem (TRO-ES-Sub) directly for each given μ and then execute a linear search of $\mu \in [0,C^{1,\mathrm{tot}}]$ to solve Top-problem (TRO-ES-Top). Table 2 shows the optimal value achieved by different schemes and the corresponding computation-time. Notice that all the results are obtained on a PC with Intel Core i5-4590 CPU@3.3GHz. As shown in Table 2, our Algorithm 4 can achieve the global optimum solution as the benchmark scheme, and, moreover, our Algorithm 4 consumes a significantly less computation-time than the benchmark scheme, which thus validates the effectiveness of our proposed algorithm.

**Figure 3:**
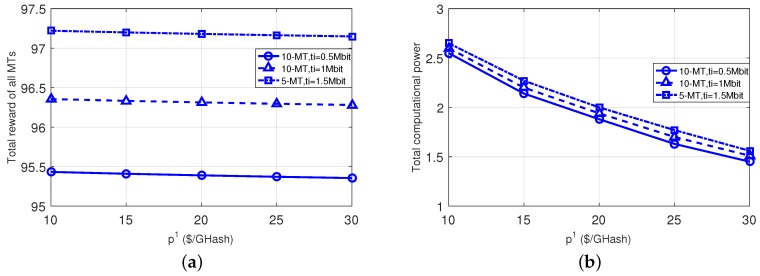
Impact of different ES prices. (**a**): total net-reward; (**b**): total computational power.

Figure 3 evaluates the impact of the ES’s price for providing the computational power to the MTs. When the price increases, the MTs become conservative in using the computational power from the ES, and thus the total computational power acquired from ES 1 decreases (as shown in the right subplot). Accordingly, all MTs’ total net-reward gradually decreases when the price increases (as shown in the left subplot).

## 4. Multi-ES Scenario and Proposed Distributed Algorithm

### 4.1. Problem Decomposition

We next consider the multi-ES scenario and focus on solving Subproblem (TRO-Sub) and Top-problem (TRO-Top). As we have described before, Problem (TRO-ES) is a non-convex optimization problem which is difficult to solve in general. To this end, we again adopt a vertical decomposition, by introducing an auxiliary variable $\nu_{i}$ which denotes MT *i*’s totally acquired computational power from all ESs, namely,

(20) $$\begin{array}{c} \nu_{i}=\sum_{k\in K}x_{i}^{k},\forall i\in I. \end{array}$$

Firstly, we assume that the values of ${\left\{\nu_{i}\right\}}_{i\in I}$ are given in advance, and we aim at solving the subproblem as follows:

(21) $$\begin{array}{cc} (\mathrm{TRO-Sub}):\;\; & H_{{\left\{\nu_{i}\right\}}_{i\in I}}^{\mathrm{sub}}=\max\sum_{i\in I}\ln\left(\left(R+rt_{i}\right)\frac{x_{i}^{\mathrm{loc}}+\nu_{i}}{\sum_{i\in I}\left(x_{i}^{\mathrm{loc}}+\nu_{i}\right)}e^{-\lambda t_{i}}-\sum_{k\in K}p^{k}x_{i}^{k}\right), \end{array}$$

(22) $$\begin{array}{cc} \mathrm{subject}\;\mathrm{to}:\;\; & 0\leq \sum_{i\in I}x_{i}^{k}\leq C^{k,\mathrm{tot}},\forall k\in K, \end{array}$$

(23) $$\begin{array}{cc} & \sum_{k\in K}x_{i}^{k}=\nu_{i},\forall i\in I,\\ \mathrm{variables}:\;\; & x_{i}^{k}\geq 0,\forall i\in I,k\in K. \end{array}$$

After solving Subproblem (TRO-Sub) and obtaining $H_{{\left\{\nu_{i}\right\}}_{i\in I}}^{\mathrm{sub}}$ (for the given ${\left\{\nu_{i}\right\}}_{i\in I}$), we continue to solve the top-problem as:

(24) $$\begin{array}{cc} (\mathrm{TRO-Top}):\; & \max H_{{\left\{\nu_{i}\right\}}_{i\in I}}^{\mathrm{sub}},\\ \mathrm{variables}:\; & 0\leq \nu_{i}\leq Q^{\max},\forall i\in I, \end{array}$$

where we set $Q^{\max}=\sum_{k\in K}C^{k,\mathrm{tot}}$.

### 4.2. Distributed Algorithm to Solve Subproblem (TRO-Sub)

The reason for us to adopt the above decomposition is that we can propose a distributed algorithm to solve Subproblem (TRO-Sub). Specifically, given ${\left\{\nu_{i}\right\}}_{i\in I}$, Subproblem (TRO-Sub) is a convex optimization problem. Thus, we again introduce the dual variable $\lambda^{k}$ to relax constraint (22) with respect to ES *k*, and obtain the corresponding Lagrangian function as:

(25) $$L\left(\left\{x_{i}^{k}\right\},\left\{\lambda^{k}\right\}\right)=\sum_{i\in I}\ln\left(\left(R+rt_{i}\right)M-\sum_{k\in K}p^{k}x_{i}^{k}\right)+\sum_{k\in K}\lambda^{k}\left(C^{k,\mathrm{tot}}-\sum_{i\in I}x_{i}^{k}\right),$$

where the fixed parameter *M* (under the given ${\left\{\nu_{i}\right\}}_{i\in i\in I}$) is:

(26) $$M=\frac{x_{i}^{\mathrm{loc}}+\nu_{i}}{\sum_{i\in I}\left(x_{i}^{\mathrm{loc}}+\nu_{i}\right)}e^{-\lambda t_{i}}.$$

An observation on (25) shows that it can be separated as follows:

(27) $$L\left(\left\{x_{i}^{k}\right\},\left\{\lambda^{k}\right\}\right)=\sum_{i\in I}L_{i}\left({\left\{x_{i}^{k}\right\}}_{\forall k\in K},{\left\{\lambda^{k}\right\}}_{\forall k\in K}\right)+\sum_{k\in K}\lambda^{k}C^{k,\mathrm{tot}},$$

where, for each MT *i*, its associated Lagrangian function is:

(28) $$L_{i}\left({\left\{x_{i}^{k}\right\}}_{\forall k\in K},{\left\{\lambda^{k}\right\}}_{\forall k\in K}\right)=\ln\left(\left(R+rt_{i}\right)M-\sum_{k\in K}p^{k}x_{i}^{k}\right)-\sum_{k\in K}x_{i}^{k}\lambda^{k},\forall i\in I.$$

Based on (28), we formulate each MT *i*’s local optimization problem as follows:

(29) $$\begin{array}{cc} (\mathrm{TRO-Sub-MT}i):\; & \left\{{\tilde{x}}_{i}^{k}\right\}=\arg\max\ln\left(\left(R+rt_{i}\right)M-\sum_{k\in K}p^{k}x_{i}^{k}\right)-\sum_{k\in K}x_{i}^{k}\lambda^{k}, \end{array}$$

(30) $$\begin{array}{cc} \mathrm{subject}\;\mathrm{to}:\; & \sum_{k\in K}x_{i}^{k}=\nu_{i},\forall i\in I,\\ \mathrm{variables}:\; & x_{i}^{k}\geq 0,\forall i\in I,k\in K. \end{array}$$

To further determine the optimal values of ${\left\{\lambda^{k}\right\}}_{k\in K}$ (i.e., the optimal solution of the dual problem), we use the following subgradient method:

(31) $$\begin{array}{c} \lambda^{k}=\max\left\{\lambda^{k}-\varepsilon \left(C^{k,\mathrm{tot}}-\sum_{i\in I}{\tilde{x}}_{i}^{k}\right),0\right\},\forall k\in K, \end{array}$$

where ε is the step-size for the dual-updating. Notice that (31) means that each ES *k* can individually update its $\lambda^{k}$ based on all MTs’ reported ${\left\{{\tilde{x}}_{i}^{k}\right\}}_{i\in I}$. Based on the above, each MT *i*’s local optimization problem (TRO-Sub-MT*i*) and each MT *k*’s dual-updating in (31), we propose the following distributed algorithm to solve Problem (TRO-Top) and Problem (TRO-Sub).

In particular, according to [45], using the diminishing step-size (in Step 7) enables us to reach the dual optimality. Thus, Algorithm 5 is guaranteed to converge to the optimal solution of Subproblem (TRO-Sub) and determine $H_{{\left\{\nu_{i}\right\}}_{i\in I}}^{\mathrm{sub}}$.

**Algorithm 5:** To solve Subproblem (TRO-Sub) and determine $H_{{\left\{\nu_{i}\right\}}_{i\in I}}^{\mathrm{sub}}$**Input**: ${\left\{\nu_{i}\right\}}_{i\in I}$. **Initialization**: Set the iteration-index l=1. Each ES k∈K initializes $\lambda^{k}\left(l\right)$. **while**
$\max_{k\in K}\left|\lambda^{k}\left(l\right)-\lambda^{k}\left(l-1\right)\right|>\varepsilon$
**do** Each ES *k* broadcasts $\lambda^{k}\left(l\right)$ to all MTs. Given ${\left\{\lambda^{k}\left(l\right)\right\}}_{k\in K}$, each MT *i* solves its local Problem (TRO-Sub-MT*i*) and obtain its ${\left\{{\tilde{x}}_{i}^{k}\right\}}_{k\in K}$. Each MT *i* reports its ${\tilde{x}}_{i}^{k}$ to each ES *k*. After collecting ${\left\{{\tilde{x}}_{i}^{k}\right\}}_{i\in I}$ from all MTs, each ES *k* updates
$$\begin{array}{c} \lambda^{k}\left(l\right)=\max\left\{\lambda^{k}\left(l-1\right)-\varepsilon \left(l\right)\left(C^{k,\mathrm{tot}}-\sum_{i\in I}{\tilde{x}}_{i}^{k}\right),0\right\},\forall k\in K, \end{array}$$
where $\varepsilon \left(l\right)=\frac{a}{b+l}$. Parameters *a* and *b* are the given values. Set l=l+1. **end while** Each MT *i* sets $\ln\left(\left(R+rt_{i}\right)M-\sum_{k\in K}p^{k}{\tilde{x}}_{i}^{k}\right)$ and reports to ES 1. ES 1 sets $H_{{\left\{\nu_{i}\right\}}_{i\in I}}^{\mathrm{sub}}=\sum_{i\in I}\ln\left(\left(R+rt_{i}\right)M-\sum_{k\in K}p^{k}{\tilde{x}}_{i}^{k}\right)$. **Output**: $H_{{\left\{\nu_{i}\right\}}_{i\in I}}^{\mathrm{sub}}$.

### 4.3. Proposed Algorithm to Solve Top-Problem (TRO-Top)

We then continue to solve Problem (TRO-Top). The difficulty in solving Problem (TRO-Top) lies in the fact that we cannot express $H_{{\left\{\nu_{i}\right\}}_{i\in I}}^{\mathrm{sub}}$ analytically for each MT *i*. To tackle this difficulty, we adopt the idea of simulated annealing (SA) [47] to determine the optimal values of ${\left\{\nu_{i}\right\}}_{i\in I}$ (which are denoted by ${\left\{\nu_{i}^{*}\right\}}_{i\in I}$). The details are shown in the following Algorithm 6. Based on the idea of SA, our Algorithm 6 executes a randomized search for finding ${\left\{\nu_{i}^{*}\right\}}_{i\in I}$:

- If the newly generated ${\left\{\nu_{i}\right\}}_{i\in I}$ (which is randomly generated within the range of the previously located ${\left\{\nu_{i}\right\}}_{i\in I}$) can improve the current best value (CBV), we then accept the newly generated ${\left\{\nu_{i}\right\}}_{i\in I}$.
- If the newly generated ${\left\{\nu_{i}\right\}}_{i\in I}$ fails to improve the CBV, we then accept it according to a certain probability, such that we can avoid being trapped by a local optimum. In particular, the probability for us to accept a non-improvement ${\left\{\nu_{i}\right\}}_{i\in I}$ gradually decreases according to an annealing process in which the annealing temperature decreases gradually. As a result, it is more likely that we will refuse to accept a non-improved ${\left\{\nu_{i}\right\}}_{i\in I}$.

Notice that, in Step 7, given the newly generated ${\left\{\nu_{i}^{\prime }\right\}}_{i\in I}$, we use Algorithm 5 to compute the value of $H_{{\left\{\nu_{i}^{\prime }\right\}}_{i\in I}}^{\mathrm{sub}}$. However, regarding our proposed Algorithm 6, we notice that it is very challenging to analytically derive its computational complexity. The key reason is due to the difficulty in deriving the complexity of the subroutine, i.e., Algorithm 5 which relies on the sub-gradient method to reach the convergence.

**Algorithm 6:** To solve Top-problem (TRO-top)**Initialization**: Initialize the temperature $T_{1}=9.9$, the decaying-rate d=0.9, the lowest temperature $T_{\mathrm{final}}=0.001$, $N_{\mathrm{count}}=0$, and the time index t=1. Randomly generate ${\left\{\nu_{i}\right\}}_{i\in I}$. Set $\mathrm{CS}={\left\{\nu_{i}\right\}}_{i\in I}$. Use Algorithm 5 to compute $H_{{\left\{\nu_{i}\right\}}_{i\in I}}^{\mathrm{sub}}$. Set $\mathrm{CV}=H_{{\left\{\nu_{i}\right\}}_{i\in I}}^{\mathrm{sub}}$. **while**$T_{t}>T_{\mathrm{final}}$**do** Set t=t+1. Randomly generate ${\left\{\nu_{i}^{{}^{\prime }}\right\}}_{i\in I}$ a plane whose central is ${\left\{\nu_{i}\right\}}_{i\in I}$. Use CVX to compute $H_{{\left\{\nu_{i}^{{}^{\prime }}\right\}}_{i\in I}}^{\mathrm{sub}}$. **if**
$H_{{\left\{\nu_{i}^{{}^{\prime }}\right\}}_{i\in I}}^{\mathrm{sub}}>\mathrm{CV}$
**then**  Set $\mathrm{CV}=H_{{\left\{\nu_{i}^{{}^{\prime }}\right\}}_{i\in I}}^{\mathrm{sub}}$.  Set $\mathrm{CS}={\left\{\nu_{i}^{{}^{\prime }}\right\}}_{i\in I}$.  Set $N_{\mathrm{count}}=0$. **else**  Generate a number ϱ according to the uniform distribution within [0,1].  **if**
$\varrho <\exp(\frac{H_{{\left\{\nu_{i}^{{}^{\prime }}\right\}}_{i\in I}}^{\mathrm{sub}}-\mathrm{CV}}{T_{t}})$
**then**   $\mathrm{CV}=H_{{\left\{\nu_{i}^{{}^{\prime }}\right\}}_{i\in I}}^{\mathrm{sub}}$.   Set $\mathrm{CS}={\left\{\nu_{i}^{{}^{\prime }}\right\}}_{i\in I}$.   Set $N_{\mathrm{count}}=0$.  **else**   Update $N_{\mathrm{count}}=N_{\mathrm{count}}+1$.  **end if** **end if** **if**
$N_{\mathrm{count}}\geq 30$
**then**  Break. **end if** Update $T_{t}=T_{t-1}*d$. **end while** **Output**: $\{\nu_{i}^{*}\}=\mathrm{CS}$ and CV as the maximum value of the objective function of Problem (TRO).

### 4.4. Numerical Results for Multi-ES Scenario

In this subsection, we show the performance of our proposed algorithms for the multi-ES scenario.

**Figure 4:**
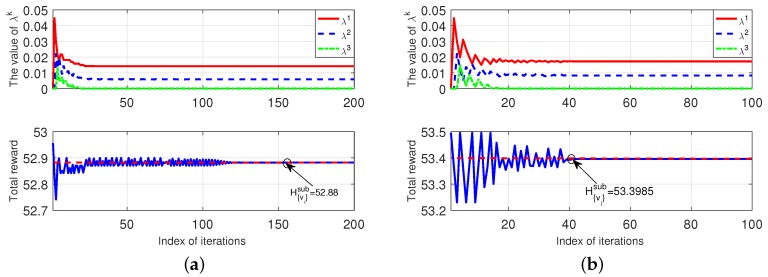
Convergence of our proposed Algorithm 5. (**a**): $\left\{\nu_{i}\right\}=\left\{1,2,3,4,5\right\}$. (**b**): $\left\{\nu_{i}\right\}=\left\{3,3,3,3,3\right\}$.

Figure 4 shows the convergence of Algorithm 5 (under the given ${\left\{\nu_{i}\right\}}_{i\in I}$). We use a 5-MT and 3-ES case, with the three ESs having $\left\{p^{1},p^{2},p^{3}\right\}=\left\{10,20,30\right\}$$/GHash. In Figure 4a, we set $\left\{\nu_{i}\right\}=\left\{1,2,3,4,5\right\}$ for the five MTs. The top-subplot in Figure 4a shows the convergence of the dual variables $\left\{\lambda^{1},\lambda^{2},\lambda^{3}\right\}$, and the bottom-subplot in Figure 4a shows the convergence of $H_{{\left\{\nu_{i}\right\}}_{i\in I}}^{\mathrm{sub}}$ to the dual optimum (which is denoted by the red-dash line). In Figure 4b, we set $\left\{\nu_{i}\right\}=\left\{1,2,3,4,5\right\}$ for the five MTs, and the results show the same convergence property as Figure 4a.

**Figure 5:**
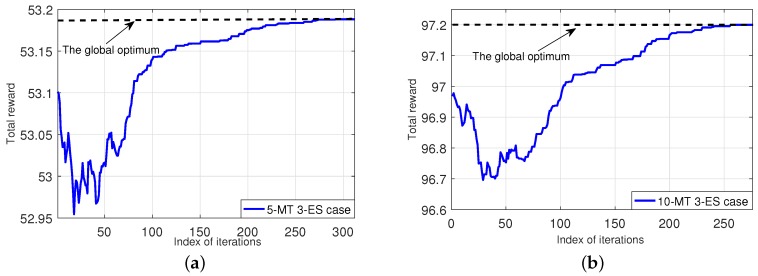
Convergence of our Algorithm 6. (**a**): a 5-MT and 3-ES case; (**b**): a 10-MT and 3-ES case.

Figure 5 shows the convergence of our Algorithm 6 for solving Top-problem (TRO-top). The left-subplot shows the case of 5-MT and 3-ES (which is used in Figure 4 before), and the right-subplot shows the case of 10-MT and 3-ES. The results show that our algorithm can quickly converge to the optimal solution (i.e., ${\left\{\nu_{i}^{*}\right\}}_{i\in I}$) and reach the global optimum of the total net-reward of all MTs.

Table 3 evaluates the accuracy and efficiency of our proposed Algorithm 6 for solving Top-problem (TRO-Top), in comparison with the exhaustive-search method. In the exhaustive-search method, we enumerate all possible ${\left\{\nu_{i}\right\}}_{i\in I}$. However, the exhaustive-search method consumes a significant computation complexity. We thus consider two cases, namely, a 5-MT and 2-ES case and a 3-MT and 2-ES case. We set $\left\{p^{1},p^{2}\right\}=\left\{10,20\right\}$$/GHash for the two ESs, and vary each MT’s block-size $t_{i}$ from 0.2 Mbit to 1 Mbit. For each tested case, we show the total net-reward (i.e., the top-value) in each cell, and the corresponding computation time (i.e., the bottom-value) in each cell. The results in Table 3 show that our Algorithm 6 achieves the optimal solution exactly the same as the exhaustive-search method, but consuming significantly less computation-time.

**Figure 6:**
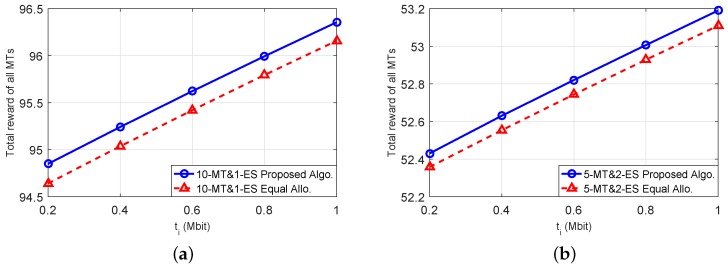
Advantage of our proposed algorithms in comparison with an Equal-Allocation scheme. (**a**): 10-MT and 1-ES case; (**b**): 5-MT and 2-ES case.

To show the advantage of our proposed algorithms, we further compare the performance of our proposed algorithms with that of a heuristic equal-allocation scheme in which each ES equally divides its total computational power to be shared by all MTs. Figure 6a below shows the results under the scenario of 10 MTs and one ES, and Figure 6b shows the results under the scenario of five MTs and two ESs. Both figures validate that our proposed algorithms can outperform the equal-allocation scheme. This advantage essentially comes from that we properly allocate the computational powers at different ESs for the MTs.

**Figure 7:**
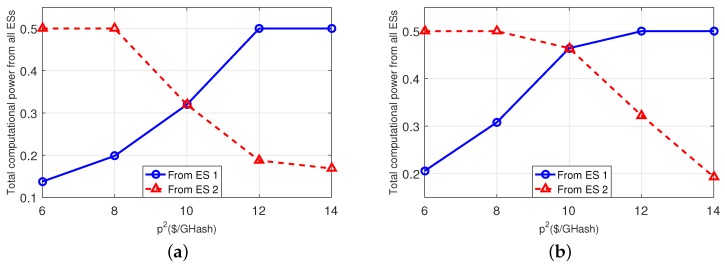
Impact of the ES prices. (**a**): 3-MT and 2-ES case; (**b**): 5-MT and 2-ES case.

Finally, in Figure 7, we evaluate the impact of the ESs’ prices for providing the computational power to the MTs. We use the same parameter-settings as Table 3, but fix $p^{1}=10$$/GHash and vary $p^{2}$ from 6 $/GHash to 14 $/GHash. Both subplots show that all MTs’ totally acquired computational power from ES-2 gradually decreases due to the increasing price. As a result, the MTs are encouraged to acquire more computational power from ES-1.

## 5. Conclusions

In this work, we have investigated the optimal computational power allocation for the multi-access MEC enabled Blockchain. In particular, we focused on the scenario in which the group of the MTs acquire the computational power from the ESs, with the objective of maximizing all MTs’ total net-reward in the mining process while keeping the fairness among the MTs. By exploiting the layered structure of the formulated optimization problem, we have proposed two distributed algorithms, namely, one for the single-ES scenario and another for the multi-ES scenario, to efficiently compute the MTs’ optimal computational power allocations. Extensive numerical results have been provided to validate the effectiveness of our proposed algorithms. In this work, we mainly focused on the reward optimization from the MTs’ perspective. For our future work, we will further consider the revenue of the ESs in providing the multi-access MEC service and investigate how different ESs adjust their prices for optimizing their revenues.

## Figures and Tables

**Table 1:** Important parameter settings.

Definition and Symbol	Value
MT *i*’s local computational power $x_{i}^{\mathrm{loc}}$	uniform distribution between 1 and 2 GHash/s
MT *i*’s block-size $t_{i}$	1Mbit
Fixed reward for each block *R*	7000$
Coefficient of variable reward *r*	1000$/Mbit
Average generating-rate of the block λ	$\frac{1}{600}$
Marginal price for computational power $p^{1}$	10$/GHash

**Table 2:** Performance of our proposed algorithm.

**5-MT Case**	$t_{i}$ **= 0.2 Mbit**	$t_{i}$ **= 0.4 Mbit**	$t_{i}$ **= 0.6 Mbit**	$t_{i}$ **= 0.8 Mbit**	$t_{i}$ **= 1 Mbit**
LS Proposed	52.4482	52.6437	52.8338	53.0190	53.1994
Computing Time	0.314 s	0.278 s	0.249 s	0.212 s	0.261 s
Benchmark	52.4482	52.6437	52.8338	53.0190	53.1994
Computing Time	148.5 s	154.4 s	140.39 s	143.2 s	146.2 s
**10-MT Case**	**$t_{i}$ = 0.2 Mbit**	**$t_{i}$ = 0.4 Mbit**	**$t_{i}$ = 0.6 Mbit**	**$t_{i}$ = 0.8 Mbit**	**$t_{i}$ = 1 Mbit**
LS Proposed	94.8489	95.2408	95.6220	95.9932	96.3549
Computing Time	0.346 s	0.352 s	0.344 s	0.331 s	0.322 s
Benchmark	94.8489	95.2408	95.6220	95.9932	96.3549
Computing Time	197.4 s	197.6 s	198.4 s	199.5 s	210.2 s

**Table 3:** Accuracy and efficiency of our Algorithm 6.

**3-MT 2-ES Case**	$t_{i}$ **= 0.2 Mbit**	$t_{i}$ **= 0.4 Mbit**	$t_{i}$ **= 0.6 Mbit**	$t_{i}$ **= 0.8 Mbit**	$t_{i}$ **= 1 Mbit**
Exhaustive Meth.	33.6742	33.7930	33.9124	34.0287	34.1356
Computing Time	889.9 s	896.2 s	913.2 s	926.2 s	917.8 s
MultiTop-Algo.	33.6742	33.7930	33.9124	34.0287	34.1356
Computing Time	93.10 s	74.93 s	108.8 s	87.29s	59.83 s
**5-MT 2-ES Case**	**$t_{i}$ = 0.2 Mbit**	**$t_{i}$ = 0.4 Mbit**	**$t_{i}$ = 0.6 Mbit**	**$t_{i}$ = 0.8 Mbit**	**$t_{i}$ = 1 Mbit**
Exhaustive Meth.	52.4315	52.6387	52.8214	53.0064	53.1970
Computing Time	1547 s	1847 s	1648 s	1788 s	1980 s
MultiTop-Algo.	52.4315	52.6387	52.8214	53.0064	53.1970
Computing Time	63.78 s	73.05 s	53.85 s	57.67 s	83.51 s
